# Effect of novel water soluble curcumin derivative on experimental type- 1 diabetes mellitus (short term study)

**DOI:** 10.1186/1758-5996-4-30

**Published:** 2012-07-04

**Authors:** Mohamed T Abdel Aziz, Mohamed F El-Asmar, Ibrahim N El-Ibrashy, Ameen M Rezq, Abdulrahman L Al-Malki, Mohamed A Wassef, Hanan H Fouad, Hanan H Ahmed, Fatma M Taha, Amira A Hassouna, Heba M Morsi

**Affiliations:** 1 Unit of Biochemistry and Molecular Biology, the Medical Biochemistry Department, Faculty of Medicine, Cairo University, Cairo, Egypt; 2 Medical Biochemistry Department, Faculty of Medicine, Ain Shams University, Cairo, Egypt; 3 Internal Medicine Department Faculty of Medicine, Cairo University, Cairo, Egypt; 4 Biochemistry Department, Faculty of Science, King Abdul-Aziz University, Jeddah, Saudi Arabia

**Keywords:** Diabetes Type 1, Heme oxygenase −1, Curcumin, Insulin secretion, Oxidative stress

## Abstract

**Background:**

Diabetes mellitus type 1 is an autoimmune disorder caused by lymphocytic infiltration and beta cells destruction. Curcumin has been identified as a potent inducer of heme-oxygenase-1 (HO-1), a redoxsensitive inducible protein that provides protection against various forms of stress. A novel water soluble curcumin derivative (NCD) has been developed to overcome low *in vivo* bioavailability of curcumin. The aim of the present work is to evaluate the anti diabetic effects of the “NCD” and its effects on diabetes-induced ROS generation and lipid peroxidation in experimental type- 1 diabetes mellitus. We also examine whether the up regulation of HO-1 accompanied by increased HO activity mediates these antidiabetic and anti oxidant actions.

**Materials and methods:**

Rats were divided into control group, control group receiving curcumin derivative, diabetic group, diabetic group receiving curcumin derivative and diabetic group receiving curcumin derivative and HO inhibitor ZnPP. Type-1 diabetes was induced by intraperitoneal injection of streptozotocin. Curcumin derivative was given orally for 45 days. At the planned sacrification time (after 45 days), fasting blood samples were withdrawn for estimation of plasma glucose, plasma insulin and lipid profile . Animals were sacrificed; pancreas, aorta and liver were excised for the heme oxygenase - 1 expression, activity and malondialdehyde estimation.

**Results:**

NCD supplementation to diabetic rats significantly lowered the plasma glucose by 27.5% and increased plasma insulin by 66.67%. On the other hand, the mean plasma glucose level in the control group showed no significant difference compared to the control group receiving the oral NCD whereas, NCD supplementation to the control rats significantly increased the plasma insulin by 47.13% compared to the control. NCD decreased total cholesterol, triglycerides, LDL cholesterol and increased HDL cholesterol levels. Also, it decreased lipid peroxides (malondialdehyde) in the pancreas, aorta and liver.

**Conclusion:**

The (NCD) by its small dose possesses antidiabetic actions and that heme oxygenase induction seems to play an important role in its anti-diabetic effects. NCD also improves the lipid profile and oxidative status directly, proved by decreasing lipid peroxides (malondialdehyde) in pancreas, liver & aorta. The new water soluble curcumin derivative still retains the essential potencies of natural curcumin.

## Background

Diabetes is the most common endocrine disorder and it was reported that by the year 2010 more than 200 million people worldwide will have diabetes mellitus (DM) and 300 million will subsequently have the disease by 2025 [1]. There is increasing evidence that complications related to DM are associated with increased oxidative stress induced by generation of free radicals [2]. These reactive oxygen species play an important role in the pathogenesis and development of complications of diabetes. The antioxidant treatment which suppresses apoptosis of β-cells was shown to preserve β-cell function in diabetic mice [3].

Exposure of endothelial cells to elevated blood glucose levels leads to the generation of excess reactive oxygen species (ROS) in the cells [4]. Hyperglycemia-mediated local formation of ROS is considered to be a major contributing factor to endothelial dysfunction, including abnormalities in cell cycling [5] and delayed replication [6]; these abnormalities can be reversed by antioxidant agents [7] or by increased expression of antioxidant enzymes [8]. Du et al. [9] demonstrated that hyperglycemia stimulates the induction of apoptosis in endothelial cells by a mechanism that involves the generation of ROS and superoxide anion (O_2_^_.^). A reduction in antioxidant reserves has been related to endothelial cell dysfunction in diabetes, even in patients with well-controlled glucose levels [10]. In a recent study [4], it was demonstrated that increased expression of heme-oxygenase −1(HO-1) attenuates glucose-mediated cell growth arrest and reduces apoptosis in human microvessel endothelial cells.

Curcumin (1, 7 bis (4 hydroxy-3 methoxy phenol)-1, 6 heptadiene-3, 5 dione) is a yellow phenolic compound present in tumeric (Curcumalonga) a widely used spice in Indian cuisine. Curcumin has a number of biological applications along with a significant antioxidant activity both invivo and invitro [11]. Studies have suggested a strong intrinsic activity and, hence, efficacy of curcumin as a therapeutic agent for various ailments. However, studies over the past three decades related to absorption, distribution, metabolism and excretion of curcumin have revealed poor absorption and rapid metabolism of curcumin that severely curtails its bioavailability [12].

Heme oxygenase (HO) catalyzes the rate-limiting step in oxidative degradation of heme to biliverdin, producing equimolar quantities of carbon monoxide (CO), iron. Biliverdin is then converted to bilirubin by bilirubin reductase. In addition, HO-1 has emerged as an important mediator of cellular defence against wide-ranging tissue injuries. The antioxidant and antiapoptotic actions of HO-1 have been also attributed to the by-products of heme degradation namely, CO and biliverdin [13].

The role of HO system in insulin release and glucose metabolism is becoming increasingly clear [14,15]. HO-mediated stimulation of insulin release has been reported in different rat strains [14]. Heme oxygenase catalyzes the carbon monoxide (CO) production, which has important biological effects, including antioxidant, anti-inflammatory, and cytoprotective functions [16]. Studies suggest a central role of CO in glucose metabolism. In the human body, CO is formed at a rate of 16.4 mol/h [17]. Normally, the islets of Langerhans produce CO and nitric oxide (NO) to regulate insulin release. While NO negatively modulates glucose-stimulated insulin release, CO stimulates insulin secretion, moreover, glucose stimulates pancreatic β-cells to produce CO, which in turn triggers insulin release [18].

The transient up-regulation of HO-1 that normally accompanies many pathophysiological conditions may represent the first line of defense against tissue injury to counteract the adverse changes that would destabilize the homeostatic in physiological milieu. This could be achieved by pharmacological agents capable of inducing HO-1 like cobalt protoporphyrin or some herbal medicines. Curcumin has been reported to induce HO-1 expression [19]. Increased HO activity is an important component in curcumin-mediated cytoprotection against oxidative stress [20].

Because of the poor bioavailability of pure curcumin, a new water soluble curcumin derivative (Patent pending PCT/EG2010/000008) was used in this study.

The aim of the present work is to evaluate the anti diabetic effects of the “NCD” and its effects on diabetes-induced ROS generation and lipid peroxidation in experimental type- 1 diabetes mellitus. We also examine whether the up regulation of HO-1 accompanied by increased HO activity mediates these antidiabetic and anti oxidant actions.

## Materials and methods

This work was performed at the Unit of Biochemistry and Molecular Biology at the Medical Biochemistry Department, Faculty of Medicine, Cairo University, Egypt. Curcumin protein conjugate was presented free of charge to the participating researchers as a personal non-profit scientific donation to help advancement of cooperation in national medical research. The novel derivative, (PCT/EG2010/000008, Published Patent Pending, WO 2011/100984) with 3.0% curcumin content is registered as international patent protected by the rights of “The Patent Cooperation Treaty” and are the personal property of its inventors.

The novel curcumin derivative “NCD” developed through covalent modification of the curcumin molecule on sites remote from its natural functional groups.

### Experimental animals

The study was carried on fifty rats, of an average weight 150–200 gm obtained from an inbred colony (Curl: HEL 1) at the Kasr Al-Aini animal experimental unit, Faculty of Medicine, Cairo University. Rats were bred and maintained in an air-conditioned animal house with specific pathogen-free conditions, and were subjected to a 12:12-h daylight/darkness and allowed unlimited access to chow and water. All the ethical protocols for animal treatment were followed and supervised by the animal facilities, Faculty of Medicine, Cairo University. All animal experiments received approval from the Institutional Animal Care Committee. The rats were divided into the following groups:

1. Twenty control rats divided into:

a) Ten Control rats were injected with citrate buffer alone to serve as a normal control group.

b) Ten rats representing the control group that receive novel water soluble curcumin derivative (NCD) (10 mg/kg body weight, was dissolved in distilled water) daily by oral gavage for 45 days.

2. Thirty diabetic rats divided into:

• Ten rats representing the streptozotocin-induced diabetic rats. (65 mg/kg of STZ dissolved in 0.1 M sodium citrate buffer, pH 4.5, intraperitoneally once)

• Ten diabetic rats that receive NCD (10 mg/kg body weight was dissolved in distilled water) daily by oral gavage for 45 days.

• Ten diabetic rats that receive NCD (10 mg/kg body weight was dissolved in distilled water) daily by oral gavage and HO inhibitor Zinc protoporphyrin (10micromol/kg, ZnPP were dissolved in sodium hydroxide 0.1 N and sodium chloride 0.9% and pH adjusted to pH 7.4) intraperitoneally once a week for 6 weeks starting from the day after development of diabetes.

### Collection of blood and tissue samples

At the planned time of sacrifaction (after 45 days), fasting blood samples (after 12–14 hrs) were withdrawn from the retro-orbital vein and processed immediately into two tubes; one containing fluoride for immediate estimation of fasting plasma glucose and the second tube containing EDTA for separation of plasma. The tubes were centrifuged and the plasma was separated and stored at – 20°C. This was followed by sacrifaction of the animals by cervical dislocation. The pancreas, aorta and liver were excised and stored at −80°C.

### Estimation of glucose and insulin

Plasma glucose was estimated by the glucose oxidase method [21] and plasma insulin was estimated [22] by a commercially available Enzyme-linked immunosorbent assay (ELISA) kit supplied by DRG Diagnostics (GmbH, Germany).

### Estimation of lipid profile

The lipid profile was estimated by available kits supplied by Diamond Diagnostics, Egypt [23-25].

### Estimation of lipid peroxides (malondialdehyde) in tissues

Pancreatic, aortic and liver tissue MDA was assayed by a commercial kit supplied by Biodiagnostic, Egypt [26].

### HO activity assay

Pancreatic, aortic and liver tissues were homogenized with 3 volume homogenization buffer. The homogenate was centrifuged at 3000 rpm for 4 min and then at 14,000 rpm for 5 min at 4°C to produce the mitochondrial pellet. The supernatant was withdrawn. The protein content was determined by the method of Lowry et al. [27]. The activity of HO in the supernatant was determined as previously described by Abraham el al. [28].

### RNA extraction

Total RNA was extracted from pancreatic, aortic and liver tissues by using SV Total RNA Isolation System supplied by Promega (Promega, Madison, WI, USA) according to the manufacturer’s protocol [29]. The yield of total RNA was determined spectrophotometrically at 260 nm, where 1 absorbance unit (A260) equals 40 μg of single stranded RNA/mL.

### Reverse transcription-polymerase chain reaction for HO-1 expression

The extracted RNA was reversed transcribed into cDNA using Access RT-PCR System kit supplied by Promega (Promega, Madison, WI, USA) following the manufacturer's instructions [30]. The cDNA products were amplified by PCR in a total volume of 50 μl containing 5 U Taq DNA polymerase (Promega) and 50 pmol each of the upstream and downstream primers. After predenaturation at 94°C for 2 min, 40 cycles were allowed to run for 30 s at 94°C, followed by 1 min at 60°C and 2 min at 68°C, and a final extension at 68°C for 7 min. Reverse transcription-polymerase chain reaction (RT-PCR) for β-actin (housekeeping gene) was performed to confirm the integrity of extracted RNA.

The primers for HO-1 (GenBank accession number J-02722) were Forward primer:5′- CTGCTAGCCTGGTTCAAGATA-3′,and Reverse primer: 5′- CATCTCCTTCCATTCCAGAG-3′. Primers for β-actin (UniSTS: 270185, PMC110200P1) were Forward primer: 5′-CCTTCCTGGGCATGGAGTCCT-3′ and Reverse primer: 5′- GGAGCAATGATCTTGATCTTC-3′.

The predicted sizes of the amplified HO-1 and β-actin DNA products were 316 bps and 202 bps, respectively. Amplified products (5 μl) were loaded onto 1.5% agarose gels previously stained with 0.5 μg/ml ethidium bromide, electrophoresed at 100 V for 30 min and then visualized by UV transilluminator. The PCR products were semiquantitated using the gel documentation system (Bio Doc Analyze) supplied by Biometra (Go¨ttingen, Germany).

### Statistical analysis

Unpaired Student's *t*-test was used for testing statistical significance of difference between every 2 groups using the PC software “Statistica version 8.0” of Statsoft Inc., USA. Data were presented as mean percent change for relevant groups. The differences between groups were considered to be significant at *p* **<** 0.05.

## Results

This study was conducted on 50 female (Curl: HEL 1) rats. At the time of scarification (after 45 days), 46 rats were remaining.

### Blood glucose and insulin levels

There was a significant increase (p < 0.001) in the mean plasma glucose level in the diabetic group (349.6 ± 77.99 mg/dL) compared to the control group (83.7 ± 6.56 mg/dL). On the other hand the mean plasma glucose level in the control group (83.7 ± 6.56 mg/dL) showed no significant difference (p > 0.05) compared to the control group receiving the oral NCD (81.9 ± 5.78 mg/dL). Oral NCD supplementation to diabetic rats resulted in a significant (27.5%) decrease (p < 0.05) in the mean plasma glucose level (253.5 ± 48.51 mg/dL) compared to the diabetic group (349.6 ± 77.99 mg/dL). There was a significant increase (p < 0.05) in the mean plasma glucose level in the diabetic group receiving the oral NCD combined with HO inhibitor ZnPP (315.9 ± 58.54 mg/dL) compared to the diabetic group receiving the oral NCD (253.5 ± 48.51 mg/dL).

A significant decrease (p < 0.001) in the mean plasma insulin level in the diabetic group (0.39 ± 0.09 μg/L) compared to control group (0.87 ± 0.24 μg/L) was detected. In addition, there was a significant increase (47.1%) (p < 0.05) in the mean plasma insulin level in the control group receiving the oral NCD (1.28 ± 0.3 μg/L) compared to the control group (0.87 ± 0.24 μg/L). Oral NCD supplementation to diabetic rats resulted in a significant increase (p < 0.001) in the mean plasma insulin level (0.65 ± 0.09 μg/L) compared to the diabetic group (0.39 ± 0.09 μg/L). Whereas, supplementation of ZnPP to diabetic rats receiving the oral water soluble curcumin showed a significant decrease (p < 0.05) in plasma insulin levels(0.52 ± 0.12 μg/L) compared to the diabetic group receiving the oral NCD (0.65 ± 0.09 μg/L) (Table 1).

**Table 1:** Biochemical parameters of studied groups

	**Plasma glucose (mg/dL)**	**Plasma insulin (μg/L)**	**Total cholesterol (mg/dL)**	**Triglycerides (mg/dL)**	**LDL cholesterol (mg/dL)**	**HDL cholesterol (mg/dL)**
**Control (n = 10)**	83.7 ± 6.56 #	0.87 ± 0.24	84.4 ± 6.85#	85.5 ± 7.56#	25.4 ± 7.82#	41.8 ± 4.18#
**Control + NCD (n = 10)**	81.9 ± 5.78 #	1.28 ± 0.3	83 ± 11.01#	83.6 ± 8.01#	25.5 ± 7.54#	40.7 ± 3.68#
**Diabetic (n = 8)**	349.6 ± 77.99*	0.39 ± 0.09	140.1 ± 30.86*	181.6 ± 25.19*	76.4 ± 26.85*	27.25 ± 4.49*
**Diabetic + NCD (n = 8)**	253.5 ± 48.51*#	0.65 ± 0.09	91.88 ± 9.61#	133.5 ± 14.74*#	25.5 ± 6.76#	38.6 ± 4.09#
**Diabetic + NCD + ZnPP (n = 10)**	315.9 ± 58.54*	0.52 ± 0.12	93.5 ± 12.51#	128.2 ± 12.09*#	27.4 ± 11.36#	40.2 ± 4.97#

Results are presented as mean ± SD.

*Values differ significantly from control (P < 0.05).

^#^Values differ significantly from diabetic p < 0.05.

NCD = novel curcumin derivative

### Malondialdehyde levels in different tissues

There was a significant increase (< 0.05) in MDA in pancreas (65.3%) (93.7 ± 12.55 nmol/g tissue), liver (55.3%) (523.7 ± 58.35 nmol/g tissue) and aorta (93.6%) (57.3 ± 7.74 nmol/g tissue) of diabetic rats compared to the corresponding control groups (56.7 ± 6.73, 337.3 ± 44.69 and 29.6 ± 5.6 nmol/g tissue), respectively.

Supplementation of the water soluble curcumin derivative to diabetic rats significantly decreased (< 0.05) the levels of MDA in these tissues (pancreas (28.9%); 66.62 ± 5.5 nmol/g tissue), (liver (29.8%; 367.7 ± 37.3 nmol/g tissue) and (aorta (19.7%); 46 ± 4.37 nmol/g tissue).

The mean MDA level in the pancreas (56.7 ± 6.73 nmol/g tissue), liver (337.3 ± 44.69 nmol/g tissue) and aorta (29.6 ± 5.6 nmol/g tissue) of the control group showed no significant difference (p>0.05) compared to the control group receiving the oral NCD (53.2 ± 8.01, 331.3 ± 33.12 and 28 ± 4.16 nmol/g tissue), respectively.

**Figure 1:**
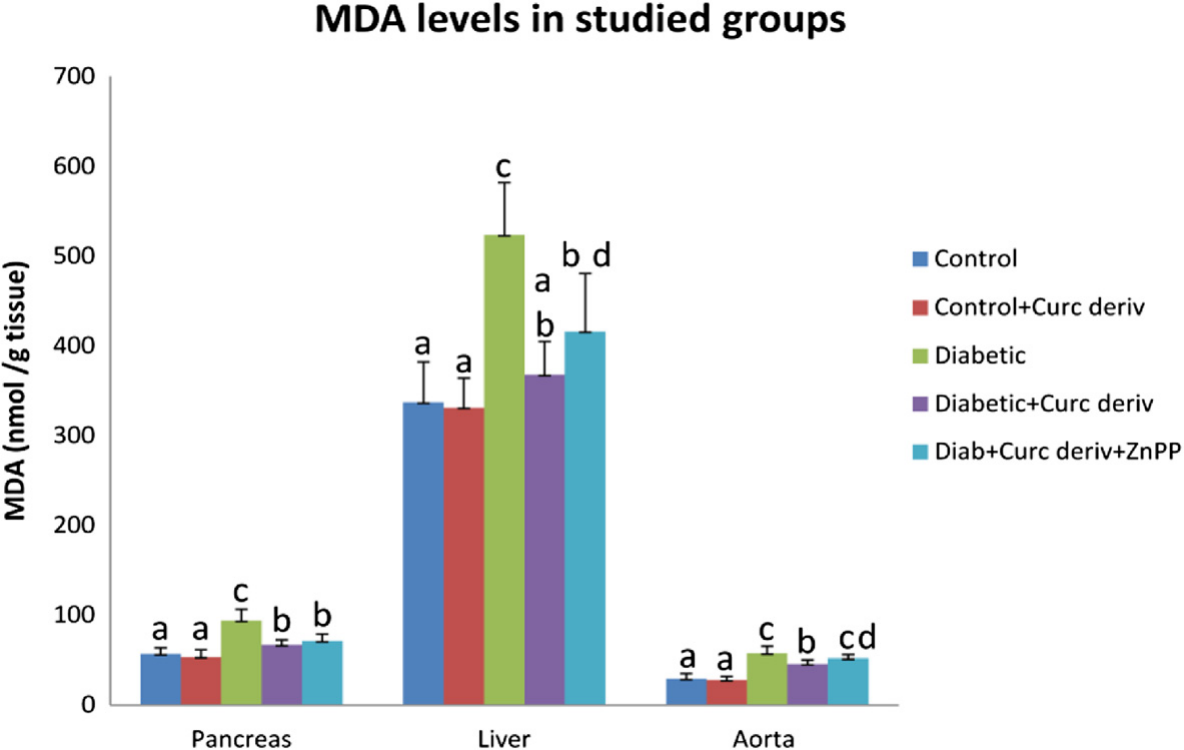
**The MDA levels (nmol/g tissue) of studied groups.** Values not sharing a common superscript differ significantly (p < 0.05). Results are presented as mean ± SD.

Although the MDA level was increased in the pancreas (70.8 ± 8.06 nmol/g tissue), liver (416.1 ± 64.2 nmol/g tissue) and aorta (51.9 ± 4.17 nmol/g tissue) of diabetic rats receiving the curcumin derivative and ZnPP compared to the diabetic rats receiving the NCD only, the increase was found to be statistically significant (p < 0.05) only in the aorta (46 ± 4.37 nmol/g tissue) (Figure 1).

### Heme –oxygenase −1 expression in different tissues

There was a significant increase (p < 0.001) in the mean HO-1 expression level in pancreas (990 ± 74.3 μg/mL) and liver (1088.3 ± 168.7 μg/mL) of diabetic rats compared to the corresponding control groups (492.7 ± 80.13 μg/mL and 560 ± 74.7 μg/mL), respectively. On the contrary, the HO-1 expression in the aorta (290.6 ± 40.7 μg/mL) of diabetic rats showed a significant decrease (p < 0.001) compared to the control group (491.4 ± 68.6 μg/mL).

Curcumin supplementation to the control rats significantly increased (p < 0.001) the HO-1 expression in the pancreas (864.3 ± 62 μg/mL), liver (852.7 ± 83.43 μg/mL) and aorta (835.9 ± 64.7 μg/mL) compared to the corresponding control groups (492.7 ± 80.1, 560 ± 74.7 and 491.4 ± 68.6 μg/mL).

In addition, curcumin supplementation to the diabetic rats significantly increased the HO-1 expression in the pancreas (1300.5 ± 166.2 μg/mL), liver (1707.7 ± 212.3 μg/mL) and aorta (758 ± 73.23 μg/mL) compared to the corresponding control groups (492.7 ± 80.13, 560 ± 74.7 and 491.4 ± 68.6 μg/mL), respectively.

**Figure 2:**
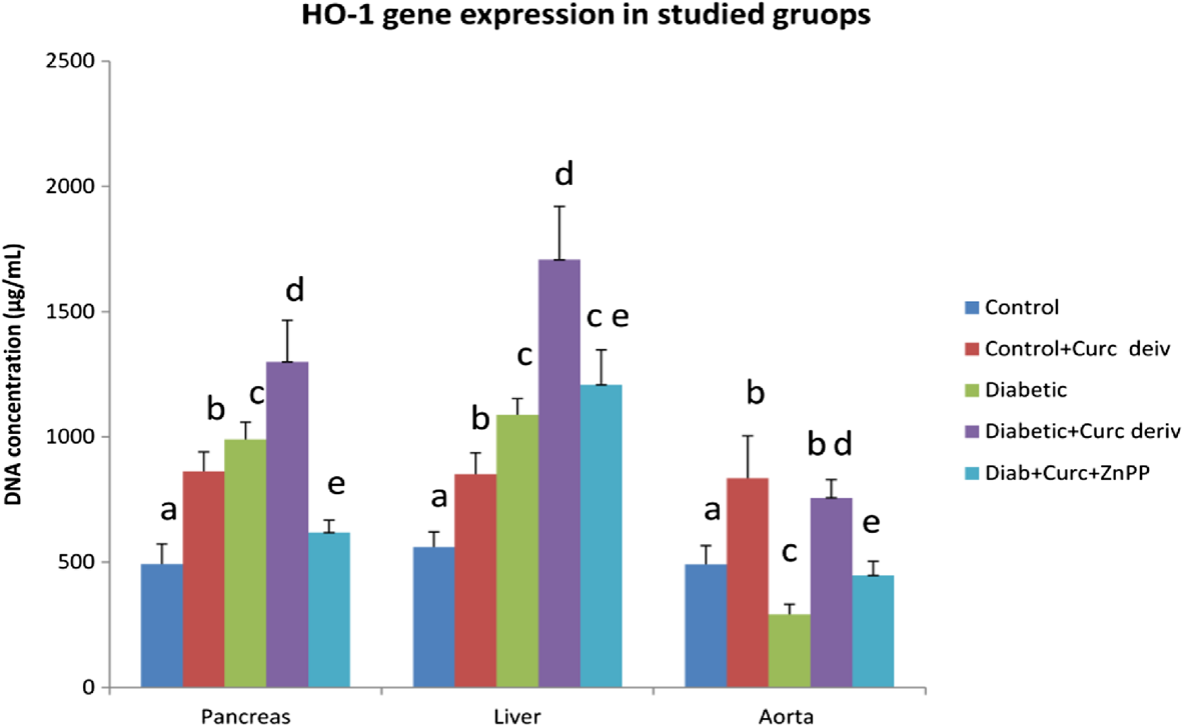
**HO-1 gene expression (μg/mL) of studied groups.** Values not sharing a common superscript differ significantly (p < 0.05). Results are presented as mean ± SD.

**Figure 3:**
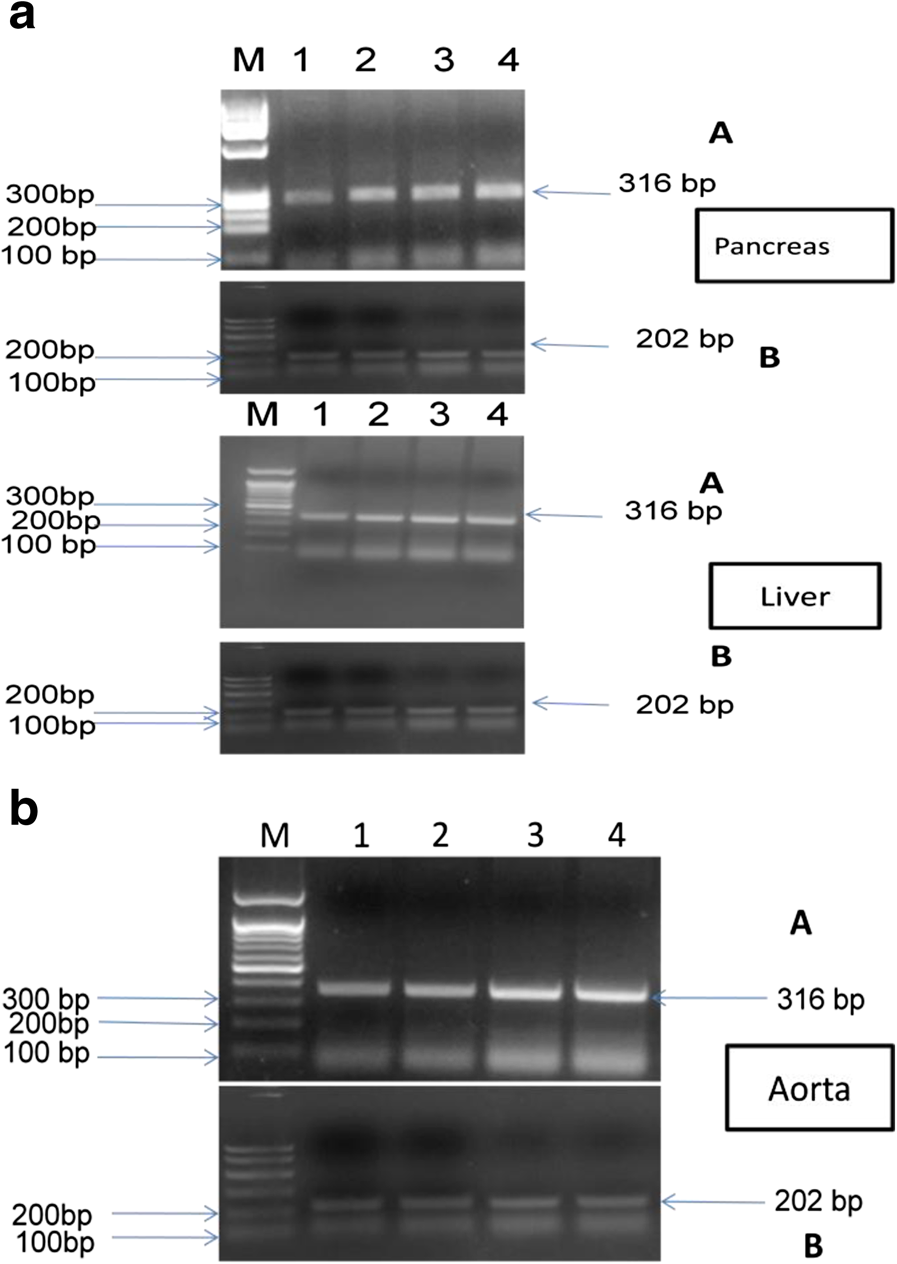
**a:Agarose gel electrophoresis showing PCR products of HO-1and β-actin genes.** Lane M: DNA marker (100,200,300 …etc.), Lanes 1–4: PCR products of (A) HO-1 gene (316 bp) and (B) β-actin gene (house keeping gene 202 bp in pancreatic and liver tissues of studied groups in order: Lane1: Control, Lane2: Control + NCD, Lane3: Diabetic, Lane4: Diabetic + NCD. **b**: Agarose gel electrophoresis showing PCR products of HO-1and β-actin genes. **b**: Agarose gel electrophoresis showing PCR products of HO-1and β-actin genes. Lane M: DNA marker (100,200,300 …etc.), Lanes 1–4: PCR products of (A) HO-1 gene (316 bp) and (B) β-actin gene (house keeping gene 202 bp in aortic tissues of studied groups in order: Lane1: Diabetic, Lane2: Control, Lane3: Diabetic, + NCD, Lane4: Control + NCD.

Also, curcumin supplementation to the diabetic rats significantly increased the HO-1 expression in the pancreas (1300.5 ± 166.2 μg/mL), liver (1707.7 ± 212.3 μg/mL) and aorta (758 ± 73.23 μg/mL) compared to the corresponding diabetic groups (990 ± 74.3, 1088.3 ± 168.7 and 290.6 ± 40.7 μg/mL), respectively (Figures 2, 3a and 3b) .

**Figure 4:**
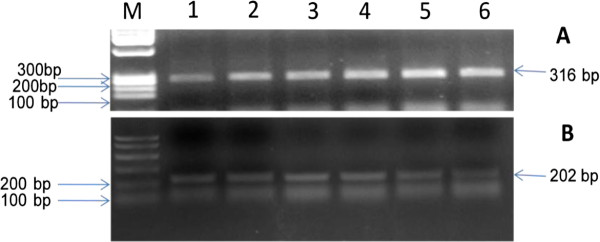
**Agarose gel electrophoresis showing PCR products of HO-1and β-actin genes.** Lane M: DNA marker (100,200,300 …etc.), Lanes 1–6: PCR products of (**A**) HO-1 gene (316 bp) and (**B**) β-actin gene (house keeping gene 202 bp in aortic, pancreatic and liver tissues of studied groups in order: Lane 1: Diabetic + NCD + ZnPP in aorta, Lane 2: Diabetic + NCD in aorta, Lane 3: Diabetic + NCD + ZnPP in pancreas, Lane 4: Diabetic + NCD in pancreas, Lane 5: Diabetic + NCD in liver, Lane 6: Diabetic + NCD + ZnPP in liver.

There was a significant decrease (p < 0.05) in the mean HO-1 gene expression in the diabetic group receiving the oral NCD combined with HO inhibitor ZnPP in pancreas (619.6 ± 49.4 μg/mL), liver (1208.4 ± 139.36 μg/mL) and aorta (446.8 ± 56.1 μg/mL) compared to the corresponding diabetic group receiving the oral NCD (1300.5 ± 166.2, 1707.7 ± 212.3and 758 ± 73.23 μg/mL), respectively (Figures 2 and 4).

### Heme –oxygenase activity in different tissues

There was a significant increase (p < 0.001) in the mean HO- activity in pancreas (1306.2 ± 182.12 pmol bilirubin/mg protein/hr) and liver (1950 ± 287.8 pmol bilirubin/mg protein/hr) of diabetic rats compared to the corresponding control groups (800 ± 184.04 pmol bilirubin/mg protein/hr and 1015 ± 213.5 pmol bilirubin/mg protein/hr), respectively. On the contrary, the HO-activity in the aorta (343.7 ± 108.35 pmol bilirubin/mg protein/hr) of diabetic rats showed a significant decrease (p < 0.001) compared to the control group (695 ± 180.2 pmol bilirubin/mg protein/hr).

Curcumin derivative supplementation to the control rats significantly increased (p < 0.001) the HO- activity in the pancreas (1040 ± 87.55 pmol bilirubin/mg protein/hr), liver (1545 ± 378.9 pmol bilirubin/mg protein/hr) and aorta (1025 ± 215.05 pmol bilirubin/mg protein/hr) compared to the corresponding control groups (800 ± 184.04, 1015 ± 213.5and 695 ± 180.2 pmol bilirubin/mg protein/hr),respectively.

In addition, curcumin derivative supplementation to the diabetic rats significantly increased the HO-activity in the pancreas (1968.7 ± 284.02 pmol bilirubin/mg protein/hr), liver (2393.7 ± 367.8 pmol bilirubin/mg protein/hr) and aorta (718 ± 106.69 pmol bilirubin/mg protein/hr) compared to the corresponding diabetic groups (1306.2 ± 182.12, 1950 ± 287.8 and 343.7 ± 108.35 pmol bilirubin/mg protein/hr), respectively.

**Figure 5:**
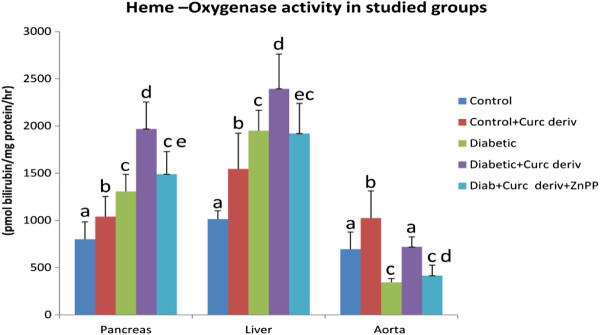
**HO- activity (pmol bilirubin/mg protein/hr) of studied groups.** Values not sharing a common superscript differ significantly (p < 0.05). Results are presented as mean ± SD.

There was a significant decrease (p < 0.05) in the mean HO-activity in the diabetic group receiving the oral NCD combined with HO inhibitor ZnPP in pancreas (1490 ± 237.81 pmol bilirubin/mg protein/hr), liver (1920 ± 318.15 pmol bilirubin/mg protein/hr) and aorta (415 ± 110.68) compared to the corresponding diabetic group receiving the oral water soluble curcumin derivative (1968.7 ± 284.02, 2393.7 ± 367.8 and 718 ± 106.69 pmol bilirubin/mg protein/hr), respectively (Figure 5).

### Lipid profile levels

The mean plasma cholesterol level, LDL cholesterol and triglycerides levels were significantly higher (p< 0.001) in the diabetic group (140.1 ± 30.86, 76.4 ± 26.85 and 181.6 ± 25.19 mg/dL, respectively) compared to the control group (84.4 ± 6.85, 25.4 ± 7.82 and 85.5 ± 7.56 mg/dL, respectively). Also, there was a significant increase (p< 0.001) in the mean plasma HDL cholesterol level in the control group (41.8 ± 4.18 mg/dL) compared to the diabetic group (27.25 ± 4.49 mg/dL).

Curcumin derivative supplementation to diabetic rats significantly decreased (p < 0.001) the mean plasma cholesterol (34.42%), LDL cholesterol (26.5%) and triglycerides (66.64%) levels (91.88 ± 9.61, 25.5 ± 6.76 and 133.5 ± 14.74 mg/dL) compared to the diabetic group (140.1 ± 30.86, 76.4 ± 26.85 and 181.6 ± 25.19 mg/dL). On the other hand, it significantly increased (p < 0.001) the plasma HDL cholesterol level (43.6%) (38.6 ± 4.09 mg/dL) compared to the diabetic group.

No significant difference (p > 0.05) was detected in all lipid profile levels between the diabetic group receiving the oral NCD and the diabetic group receiving the oral water soluble curcumin derivative combined with HO inhibitor ZnPP (Table 1).

## Discussion

To date, most approaches to reduce oxidative stress in human diabetes have met with little success, and it is now evident that alternative approaches are required. There are efforts to search for alternative drugs and herbal medicine that will be a new choice in diabetic treatment.

Of those is curcumin which is a naturally occurring yellow pigment powder extracted from the roots of *Curcuma longa*. The potential beneficial effects of curcumin have been shown to exhibit anti-inflammatory [31], antioxidant [32], anti-tumor [33] and anti-diabetic activities [34].

In spite of its efficacy and safety, curcumin has the major problem of low bioavailability [12]. The present work evaluates the effect of a novel curcumin derivative (NCD) developed to overcome the low bioavailability of curcumin, (PCT/EG2010/000008, Published Patent Pending, WO 2011/100984). The NCD was designed to contain the equivalent of only 3.0% curcumin. Induction of D.M. led to a significant increase of plasma glucose level and a significant decrease in plasma insulin as compared to the normal control group. Treatment with the NCD showed a non-significant decrease in plasma glucose with a significant increase in plasma insulin in the control group that receive NCD, as compared to the normal control group while it significantly lowered the plasma glucose and increased plasma insulin in the NCD diabetic group, as compared to the diabetic group.

Several studies proved the effect of curcumin and/or its derivatives on lowering blood glucose levels in STZ-induced D.M and in diabetic db/db mice [34,35]. Rungseesantivanon et al., (2010) [36] tested the effect of curcumin in 30 and 300 mg/Kg body weight doses for six weeks on STZ-induced diabetic rats. They reported that both doses of curcumin significantly lowered blood glucose by 18.73% and 32% respectively, in the diabetic but not in the control groups. Also Wickenberg et al., (2010) [37] reported that the ingestion of 6 g Curcuma longa increased postprandial serum insulin levels, but did not affect plasma glucose levels in healthy subjects. The results indicate that Curcuma longa may have an effect on insulin secretion. They pointed to the fact that in healthy subjects, glucose levels are strictly regulated, and it is difficult to measure differences in plasma glucose levels. This could explain why we did not see any significant differences in plasma glucose levels.

EL-Azab et al., (2011) [38] reported that intraperitoneal injection of curcumin (10 mM. 100μL/mouse) for 28 days showed a significant reduction in blood glucose in STZ-induced D.M but failed to return to the normal levels. Curcumin also significantly increased the insulin level, yet still to a lower extent than the normal levels.

On the other hand, Best et al., (2007) [39], reported that curcumin induced electrical activity in rat pancreatic β-cells by activating the volume-regulated anion channel *f*, with depolarization of the cell membrane potential, the generation of electrical activity, and enhanced insulin release. Another report indicated that curcumin treatment enhances islet recovery by inducing heat-shock protein Hsp70, a response protein, during cryopreservation (Kanitkar and Bhonde, 2008) [40] so the activation of β-cells function by curcumin might contribute to the hypoglycemic actions of this compound.

This agrees with the results of our previous work on the effect of curcumin on insulin release in rat isolated pancreatic islets. We reported that insulin secretion was significantly increased in islets incubated with curcumin. Insulin secretion was significantly decreased by incubation of islets with stannus mesoporphyrin (HO activity inhibitor), indicating the role of HO-1 in insulin secretion in pancreatic islets [41]. Additionally, curcumin was found to induce HO-1 expression, which has been reported to have cytoprotective effects in mouse pancreatic β-cells as mediated through the activation of NF-E2-related factor 2 (Nrf2) and phosphotidylinositol 3-kinase (PI3-kinase)/Akt-mediated signaling [42].

As for hyperlipidemia it is an associated complication of D.M. Significant changes in the lipid profile due to induction of D.M. led to, elevations in serum triglycerides, cholesterol and LDL with a significant decrease in serum HDL compared to the normal control group. Treatment with the NCD showed non-significant changes in serum triglycerides, cholesterol LDL and HDL in the NCD control group as compared to the normal control group. Respective significant decreases were found in the NCD diabetic group which gained a significant increase in HDL as compared to the diabetic group.

Kim and Kim, (2010) [43] reported that curcumin significantly decreased serum triglyceride, cholesterol and LDL and enhanced hepatic cholesterol 7α-hydroxylase (CYP7A1) gene expression, a rate limiting enzyme in the biosynthesis of bile acid from cholesterol at the mRNA level, which may partially account for the role in the cholesterol lowering effect of curcumin. Accordingly, we can suggest that curcumin exerts its cholesterol-lowering actions by modulating cholesterol degradation, rather than through an antioxidant mechanism.

In D.M. hyperglycemia is thought to induce cell death through free radical formation [44]. Elevated generation of free radicals may lead to oxidative damage to cell membrane and enhance lipid peroxidation [45]. The most commonly used indicator of lipid peroxidation is MDA [46].

### High doses of cell toxins like streptozotocin and alloxan induce insulin deficiency and type

1 diabetes mellitus with ketosis [47]. Several markers of vascular inflammation have been shown to be influenced by the presence of ketosis. It has been reported that acetoacetate, but not β-hydroxybutyrate, increases lipid peroxidation and growth inhibition in cultured human endothelial cells [48], as well as lowering glutathione levels in human erythrocytes [49].

In the present study MDA reflected the state of diabetic lipid per-oxidation in the pancreas, liver and aorta as there was significant increase in MDA in the pancreas as compared to the normal control group. Treatment with the NCD significantly decreased the levels of MDA in the pancreas, liver, and aorta. Meanwhile, the same treatment showed non-significant decrease in MDA levels in the respective tissues of the NCD control group.

Suryanarayana et al., (2007) [50] reported increased lipid peroxidation in the liver, kidney, pancreas and heart of diabetic rats. Previous report [51] showed that curcumin increased hepatic reduced glutathione (GSH) and induced GSH transferase to prevent lipid peroxidation and detoxify toxic lipid aldehydes in diabetic rats, while another study [52] showed that curcumin inhibits protein glycosylation, lipid peroxidation, and oxygen radical generation in human red blood cells exposed to high glucose levels.

In the present study paralleled significant increases in the expression of HO-1 and HO activity due to induction of D.M. was found in the pancreas and liver with significant decrease in aorta. Meanwhile, the treatment has led to a significant increase HO-1 expression in the NCD control group in the respective tissues with significant paralleled increases in HO-activity. Treatment of the diabetic group led to significant increases in expression of HO-1 with significant paralleled increases in HO-activity.

To determine whether curcumin's action is mediated *via* inducible HO-1, the HO inhibitor (ZnPP) was administered to diabetic rats receiving oral curcumin derivative. Administration of ZnPP to diabetic rats receiving curcumin derivative showed a significant increase in the plasma glucose level and a significant decrease in insulin levels when compared to the diabetic group and diabetic group receiving curcumin derivative only. This suggests that the hypoglycemic action of curcumin may be, in part, mediated through HO-1 induction.

The decrease in HO-1 expression and activity in aortic tissue in the present work agrees with the work of Abraham et al., (2004) [53] who reported that the decrease in HO-1 gene expression and activity in aortic tissues observed with hyperglycemia in experimental diabetes is associated with an increase in shedding of endothelial cells into the circulation, presumably reflecting cell damage.

Although the MDA level was increased in the pancreas, liver and aorta of diabetic rats receiving the curcumin derivative and ZnPP compared to the diabetic rats receiving the curcumin derivative only, the increase was found to be statistically significant (p < 0.05) only in the aorta . Thus indicating that curcumin decreased the MDA levels in pancreatic and hepatic tissues by a direct effect through acting on other antioxidant enzymes while in aortic tissue this decrease in MDA level might be through HO-1.

## In conclusion

The water soluble curcumin derivative (NCD) by its small dose with 3.0% curcumin content only has the ability to decrease plasma glucose and increase plasma insulin levels significantly in diabetic rats. We find that antidiabetic actions exerted by NCD may be linked to heme oxygenase function as a crucial defensive and detoxifying cellular system, so, heme oxygenase induction seems to play an important role in its anti-diabetic effects. NCD appears to improve the lipid profile in diabetic rats by lowering total cholesterol, LDL, and triglycerides, while raising HDL levels. It is believed that curcumin exerts its cholesterol-lowering actions by modulating cholesterol absorption, degradation, or elimination, rather than through an antioxidant mechanism. NCD also improves oxidative status, protects and enhances endogenous defenses directly proved by decreasing lipid peroxides (malondialdehyde) in pancreas & liver. The new water soluble curcumin derivative still retains the essential potencies of natural curcumin.

### Recommendations

NCD (given in small doses) provides an opportunity to expand the clinical range of this efficient herbal agent by enabling its water solubility. Future studies utilizing water soluble derivative are recommended to be studied in humans.

## Competing interests

The authors declared no conflicts of interest with respect to the authorship and/or publication of this article.

## Authors’ contributions

MT contributes in study design, manuscript drafting and critical discussion. MF contributes in study design, and critical discussion.IN contributes in study design in analysis and manuscript drafting.AR contributes in preparation of the novel curcumin derivative and statistical analysis. AA contributes in preparation of the novel curcumin derivative and manuscript drafting.MA contributes in analysis and manuscript drafting.HHA contributes in study design, practical work, manuscript drafting and critical discussion.HHF contributes in analysis andmanuscript drafting.FM contributes in practical work, manuscript drafting and critical discussion. AA contributes in practical work, manuscript drafting and critical discussion. HM contributes in practical work, manuscript drafting and critical discussion. All authors read and approved the final manuscript.
